# Level of knowledge, and risk perception of mpox disease among primary healthcare workers in Nigeria: a cross-sectional study of Jigawa, Oyo and Lagos States

**DOI:** 10.11604/pamj.2024.48.176.42913

**Published:** 2024-08-14

**Authors:** Damola Bakare, Julius Salako, Abiodun Sogbesan, Omotayo Emmanuel Olojede, Kofoworola Olamide Akinsola, Rami Subhi, Hamish Graham, Adegoke Falade, Carina King, Ayobami Adebayo Bakare

**Affiliations:** 1 Department of Paediatrics, University of Ibadan, Ibadan, 200005, Nigeria,; 2 Centre for International Child Health, Murdoch Children's Research Institute, University of Melbourne, Royal Children's Hospital, Parkville, Victoria, 3052, Australia,; 3 Department of Paediatrics, University College Hospital, Ibadan, 200221, Nigeria,; 4 Department of Global Public Health, Karolinska Institutet, Stockholm, 17177, Sweden,; 5 Department of Community Medicine, University College Hospital, Ibadan, 200221, Nigeria

**Keywords:** Knowledge, monkeypox, perception, communicable, control, disease, transmission, risk-assessment, healthcare, Nigeria

## Abstract

**Introduction:**

in Nigeria, studies on mpox among primary healthcare workers are scarce despite increasing incidence of mpox disease between 2017-2022. This study aimed to assess primary healthcare workers knowledge and perception of mpox in Nigeria.

**Methods:**

we conducted a cross-sectional survey among primary healthcare workers in Nigeria (Oyo, Lagos, and Jigawa) to represent different health system capacities and socio-economic contexts. Knowledge of mpox was evaluated in four domains: general knowledge, transmission, signs and symptoms, and prevention and treatment. Each correct response received a score of 1. We categorize the level of knowledge based on the score using the mean score as the cut-off by re-classifying the composite score of respondents for each state into a binary outcome of “good knowledge” if the mean composite score was greater or equals to the mean of overall knowledge score for the three states (16.1), and “poor knowledge” if the mean score equals to sixteen or less than sixteen (≤16). Factors associated with mpox knowledge were explored using multivariable logistic regression at a 5% significance level. Perception of mpox was assessed using five constructs from the health belief model, measured on 3-point Likert scales. Factors associated with each construct were analyzed using Kruskal-Wallis and Mann-Whitney-U tests.

**Results:**

in our study on healthcare workers, 78.3% (n=239) were aware of mpox disease. Their overall knowledge was moderate, particularly regarding transmission. Meanwhile, less than 50% knew mpox can be transmitted through sharing utensils, and 65.3% (n=156) understood contact with infected animals could lead to transmission. Lagos had lower overall knowledge scores (15.3±2.3) compared to Jigawa (16.9±2.3) and Oyo (16.3±2.5) (p<0.001). Perceived susceptibility was similar across states (p=0.127), and 97.5% (n=233) believed mpox can affect anyone, while 47.3% (113) felt they couldn't contract it. Jigawa exhibited higher perceived severity (p<0.001) and barriers to prevention (p<0.001).

**Conclusion:**

primary healthcare workers in all settings had limited knowledge of mpox transmission, with the perception of mpox varying by state and participants' socio-economic characteristics. The responsibility of HCW encompasses a range of activities that include diagnosis, patient care and education, and public health interventions amongst others. Hence it is important to educate HCWs on mpox disease to successfully curtail the spread of mpox.

## Introduction

Monkeypox (Mpox) is a viral disease that belongs to the family of Poxviridae viruses - large, complex DNA viruses, and it is characterized by two strains with varying case fatality rates [1]. More than 87,000 cases and 141 deaths attributed to human mpox were reported in 111 countries worldwide between January 1, 2022 and February 1, 2023 [2,3]. Mpox has been reported in central and western African countries, like Cameroon, Central African Republic, Democratic Republic of Congo, and Nigeria by the WHO since 1970 [4-7], but the true burden of mpox in sub-Saharan Africa remains poorly defined. The increased global transmission of mpox in 2022 underscores the need for increased surveillance and public health emergency preparedness, most especially in settings with weak health systems, like Nigeria [7-9].

In Nigeria, 762 mpox confirmed cases and seven deaths were reported in 2022 (CFR: 0.9%), surpassing the cumulative number of cases reported in the previous five years [10]. A previous study conducted in Nigeria highlighted the severity of mpox and associated mortality among individuals co-infected with Human Immunodeficiency Virus (HIV) and the decline in community immunity resulting from the waning coverage of smallpox vaccination [11] (which granted some cross-protection as mpox and smallpox are orthopoxviruses [12]). Diagnosis of mpox without laboratory testing is challenging, as it shares clinical similarities with other illnesses such as chicken pox and measles. The surge in incidence of mpox, as well as emerging and re-emerging diseases like Ebola virus disease, Lassa fever, Anthrax and Marburg virus, which are zoonotic and/or epidemic prone diseases, underlines the need for greater attention to these categories of diseases [13,14].

The primary mode of mpox transmission to humans is via direct contact with infected animals or close contact with body fluid from infected persons [7,15]. In regions where mpox is not endemic, the risk of exposure to infected animals or persons is low, which may result in a lack of awareness and low index of suspicion among healthcare workers. Adequate knowledge of healthcare workers is important for early detection and effective public health response, including rumour management and promoting care seeking behaviours among community members.

Previous research in Nigeria has shown that healthcare workers have limited knowledge about the mpox virus, its nature, mode of transmission, and prevention mechanisms [16]. These studies have been online surveys and not specifically focused on primary healthcare workers who are closer to the community members [17,18]. Understanding the knowledge and risk perception of primary healthcare workers regarding mpox in therefore important for mpox outbreak preparedness and response in Nigeria. We therefore aimed to access the knowledge and risk perceptions of primary healthcare workers in different geopolitical and socio-cultural context in Nigeria.

## Methods

**Study design and settings:** we conducted a cross-sectional study with healthcare workers in three states in Nigeria, Oyo and Lagos and Jigawa, from August 15, 2022, to November 30, 2022. We aimed for geographical and socio-economic diversity in the selection of these states, while leveraging our existing research networks in these areas unrelated to mpox [19,20]. Oyo and Lagos States are primarily inhabited by the Yoruba ethnic group, with Christianity and Islam being the dominant religions while Jigawa State is predominantly inhabited by the Hausa and Fulani ethnic groups and Islam is the predominant religion. Jigawa is also the least populated state of the three. According to the National Population Estimates for 2016, with an estimated population of 5.8 million people compared to Oyo and Lagos which have estimated population of 7.8 million and 12.6 million people respectively [21]. There is a wide disparity in wealth distribution among the surveyed states too. In Jigawa state, just 1.3% of the population are within the richest wealth quantile, compared to 26.2% and 71.9% in Oyo and Lagos states respectively [22]. Our study was carried out in Lagelu and Ibadan southwest Local Government Area (LGA) in Oyo State, Kiyawa and Dutse LGAs in Jigawa state, and Ikorodu LGA in Lagos State.

**Study population:** the data was collected from healthcare workers, inclusive of all cadres (including midwives and CHEWs) who provide clinical services in primary health facilities in the selected LGAs, excluding those that are not directly involve in medical care (like cleaners and security guards). We decided to focus on primary care healthcare workers due to their proximity to community members and their crucial roles in providing health promotion and preventive services. By targeting this group, the research aimed to gather insights from those directly involved in healthcare delivery at the grassroots level. The sample size calculation was based on the formula for estimating a proportion:


$$n=Z\alpha^{2}\left(P\left(1-P\right)\right)/d^{2}$$

Where p, the level of awareness of mpox which is taken as 0.92 based on study conducted in Indonesia among healthcare workers [23]. Zα represents the z-score at 95% confidence interval (z=1.96), and d is the precision (of 5% (d=0.05). This required 113 respondents, which we increased to 124 respondents per state (372 in all the three states), to allow for 10% non-response. We thereafter corrected for finite population, given that the number of primary healthcare workers is less than 10,000 in each state [24]. We assumed 1250 eligible primary healthcare workers, reducing the sample size to 287 (96 per state).

**Data collection:** data was collected using an interviewer-administered questionnaire. The data collectors had a minimum secondary education and were proficient in English language and local dialects (Yoruba, Hausa/Fulani languages) The training of data collectors was for 6 days, including 3-days of in-class teaching through teleconferencing and 3-days field practice. Thereafter, the study tool was pre-tested for 2 weeks with community members. Data was collected on Android tablets using Open Data Kit (ODK). Each selected facility was visited 1-3 times and data collectors administered questionnaires to all consented healthcare workers who were present at the time of visit. For multiple visits, we assigned same data collectors to the same facilities and asked the respondents if they had earlier participated in the study to minimize duplicating interviews. In each selected LGA, we identified a list of primary health facilities registered from the government database and randomly selected 10 functional primary health centres in each LGA excluding health posts. Overall, we included 50 primary health centers. We identified HCWs by convenience sampling, including those who were: (1) providing medical or preventive services in the selected facilities, (2) at least 18 years old, and (3) able to communicate in English language or the predominant local dialects (Hausa, Fulani and Yoruba). All healthcare workers on clinical duties during visit by the data collectors were approached for participation. To ensure data quality, the research team conducted bi-weekly meetings and performed periodic data cleaning and verification. Any queries or concerns were promptly addressed.

**Definitions:** the primary outcome of interest was awareness of mpox, assessed through the question “Have you heard about monkeypox before?”. Secondary outcomes were knowledge and risk perception of mpox among healthcare workers. We assessed knowledge across four domains: (1) general knowledge (n=2), (2) transmission (n=6), (3) signs and symptoms, (n=5) and (4) prevention and treatment (n=8). Knowledge questions were based on information provided on mpox by the World Health Organization (WHO) and the Nigeria Centre for Diseases and Control (NCDC) [15].

**Data management and statistical analyses:** we used a scoring system based on the National Monkeypox Public Health Response Guidelines [15], to score participants' knowledge of monkeypox, assigning a maximum score of 21 based on correct responses. Respondents' composite scores were categorized into “good knowledge” if their mean score surpassed the overall mean (16.1) of all respondents and “poor knowledge” if it equaled or fell below sixteen. We report summary statistics for respondents' socio-demographic characteristics, awareness, and source of information about monkeypox using frequencies and percentages. One-way analysis of variance was used to compare the mean scores between the states, followed by univariable analyses examining the association between each covariate and “good knowledge,” presented as unadjusted odds ratios with 95% confidence intervals. This was followed by multiple logistic regression analysis to assess the association between all respondent's socio-demographic characteristics and “good knowledge” of mpox in a single model. We assessed perception of mpox based on 5 constructs from the health belief model: perceived susceptibility, perceived severity, perceived benefits, perceived barriers, and self-efficacy [25]. We asked questions to assess respondents' perception under each construct. A score of 1 was given to the response which points to the focus of each construct. We then calculated an aggregate score for each construct and used Kruskal-Wallis and Mann-Whitney U test to assess associated factors where appropriate. Any missing demographic data were reported missing at the foot of Table 1.

**Table 1:** demographic characteristics of respondents (N=305)

Respondent variables		Jigawa (N=113)	Lagos (N=93)	Oyo (N=99)
		n (%)	n (%)	n (%)
Age*	15-29 years	54 (47.8)	25 (26.9)	28 (28.3)
	30-44 years	46 (40.7)	53 (57.0)	35 (35.4)
	45 years and above	12 (10.6)	15 (16.1)	14 (14.1)
Highest level of education	Primary	5 (4.4)	0 (0.0)	0 (0.0)
	Secondary	2 (1.8)	10 (10.7)	13 (13.1)
	Tertiary/further	106 (93.8)	83 (89.3)	86 (86.9)
Ethnicity	Yoruba	2 (1.8)	85 (91.4)	96 (97.0)
	Hausa/Fulani	111 (98.2)	8 (8.6)	3 (3.0)
Marital status	Never married	64 (56.6)	71 (76.3)	67 (67.7)
	Ever married	49 (43.4)	22 (23.6)	32 (32.3)
Religion	Christianity	5 (4.4)	72 (77.4)	70 (70.7)
	Islam	108 (95.6)	21 (22.6)	29 (29.3)
Gender	Male	56 (49.6)	10 (10.7)	11 (11.1)
	Female	57 (50.4)	83 (89.3)	88 (88.9)
Cadre	CHEW/CHO	66 (58.4)	44 (47.3)	50 (50.5)
	Nurse/Midwife	2 (1.8)	17 (18.3)	23 (23.2)
	Others	45 (39.8)	32 (34.4)	26 (26.3)
Average monthly income	Less than 30,000	56 (49.6)	47 (50.5)	9 (9.1)
	30,000 and above	53 (46.9)	26 (27.9)	8 (8.1)
	No response	4 (3.5)	20 (21.5)	82 (82.8)
Awareness of monkeypox**	Yes	80 (70.8)	83 (89.2)	76 (76.8)
	No	33 (29.2)	10 (10.7)	21 (21.2)
		**n=80**	**n=83**	**n=76**
Sources of information	Radio/Television	43 (53.7)	25 (30.1)	36 (47.4)
	School	8 (10.0)	6 (7.2)	11 (14.5)
	Facility/NGO	6 (7.5)	29 (34.9)	6 (7.9)
	Online/social media	18 (22.5)	15 (18.1)	21 (27.6)
	Others	5 (6.3)	8 (9.6)	2 (2.6)

* Missing respondent age (n=23) ** Missing awareness of mpox (n=2)

**Ethical considerations:** this study adhered to the principles outlined in the Helsinki Declaration and the Nigerian National Code of Health Research Ethics. Ethical approval was obtained from the relevant authorities, including the Jigawa State Government (ref: JGHREC/2022/110), Oyo State Ministry of Health (ref: AD/13/479/44533), and Lagos State Government (LREC/06/10/2022). Prior to participation, verbal consent was obtained from all participants, who were also given the opportunity to review the informed consent form.

## Results

**Figure 1:**
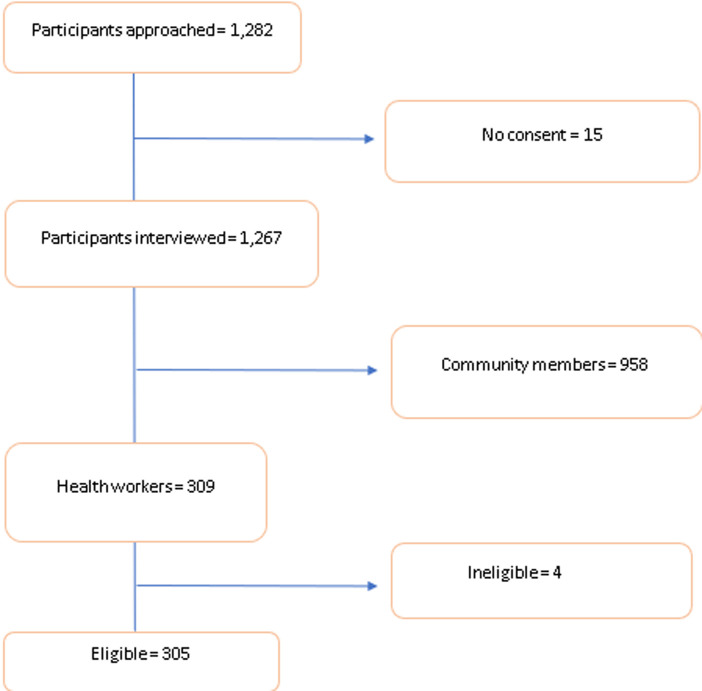
participant inclusion flow diagram

**General characteristics of the study population:** in total, we recruited 305 healthcare workers, from 50 facilities that were sampled from the five LGAs across the three states. Table 1 shows a summary of the healthcare workers' characteristics. Ninety point two percent (90.2% (275)) of the healthcare workers had tertiary level of education. Ninety-eight point two percent (98.2% (111)) in Jigawa Hausa/Fulani, while 91.4% (85) in Lagos and 97.0% (96) in Oyo state were of Yoruba ethnicity. Additionally, 58.4% (66) of those recruited in Jigawa were CHEW/CHO, compared to 47.3% (44) in Lagos and 50.5% (50) in Oyo state. Overall, 78.4% (239) healthcare workers reported they were aware of mpox. The major sources of information were radio/television (104/239) and online/social media (54/239) (Table 1) (Figure 1).

**Knowledge and perception of mpox among primary healthcare workers:** knowledge and perception of mpox among primary healthcare workers in Nigeria were relatively consistent across three states, with 96.6% (n=231) aware of its infectious nature and zoonotic transmission. However, knowledge of mpox transmission was notably lower compared to knowledge of its symptoms and methods of prevention and treatment. Only 33.9% (n=81) recognized utensil sharing as a transmission risk, and 65.3% (n=156) understood animal contact as a mode of transmission. Mean knowledge scores significantly differed between states (Jigawa 16.9, Lagos 15.3, Oyo 16.3) (p<0.001), with variations in transmission and prevention/treatment knowledge. While 97.5% (n=233) believed anyone could contract mpox, only 47.3% (n=113) believed in their own susceptibility. Despite 95.8% (n=229) perceiving mpox as severe, more Jigawa healthcare workers (77.5%, n=62) believed it could be fatal compared to Lagos (47.0%, n=39) and Oyo (42.1%, n=32) (Table 2).

**Table 2:** knowledge and perception of mpox among primary health care workers (N=239)

Knowledge of mpox	Jigawa(N=80)	Lagos (N=83)			Oyo (N=76)						Total (N= 239)
	n (%)	n (%)			n (%)						n (%)
**General knowledge (selected yes)**											
Animal-Human transmission	78 (97.5)	79 (95.2)			74 (97.4)						231(96.6)
monkeypox is caused by an infective organism	76 (95.0)	80 (96.4)			75 (98.7)						231 (96.6)
**Knowledge of transmission (selected true)** monkeypox can be transmitted through											
Sharing of clothing, towel and bedding with infected person	57 (71.2)	47 (56.6)			49 (64.5)						153 (64.0)
Sitting in the same vehicle with infected person	34 (42.5)	15 (18.1)			37 (48.7)						86 (36.0)
Contact with body fluid of infected person	59 (73.8)	64 (77.1)			65 (85.5)						188 (78.7)
Contact with infected animal	71 (88.7)	42 (50.6)			43 (56.6)						156 (65.3)
Sexual intercourse with infected person	36 (45.0)	31 (37.4)			36 (47.4)						103 (43.1)
Sharing utensils with an infected person	32 (40.0)	17 (20.5)			32 (42.1)						81 (33.9)
**Knowledge of signs and symptoms (selected yes)**											
Fever	77 (96.3)	80 (96.4)			74 (97.4)						231 (96.6)
Headache	65 (81.3)	73 (88.0)			70 (92.1)						208 (87.0)
Back pains	49 (61.3)	47 (56.6)			53 (69.7)						149 (62.3)
Swollen lymph nodes	57 (71.2)	73 (87.9)			67 (88.2)						197 (82.4)
Rashes	77 (96.3)	83 (100.0)			72 (94.7)						232 (97.1)
**Knowledge of prevention and treatment (selected true)**											
Avoiding consumption of bushmeat	78 (97.5)	61 (73.5)			65 (85.5)						204 (85.4)
Disinfecting object regularly	77 (96.3)	72 (86.7)			67 (88.2)						216 (90.4)
Proper cooking of animal product	74 (92.5)	67 (80.7)			65 (85.5)						206 (86.2)
Following the healthcare worker's advice is important if I suspect that I have monkeypox	80 (100.0)	80 (96.4)			75 (98.7)						235 (98.3)
People with monkeypox should avoid scratching their skin	76 (95.0)	72 (86.7)			67 (88.2)						215 (89.9)
People with monkeypox should have enough sleep	53 (66.3)	57 (68.7)			49 (64.5)						159 (66.5)
monkeypox may resolve spontaneously	7 (8.8)	11 (13.3)			15 (19.7)						33 (13.8)
There is an available vaccine for monkeypox	18 (22.5)	24 (28.9)			18 (23.7)						60 (25.1)
**Knowledge scores**	Jigawa state	Lagos state			Oyo state				Total		P-value^h^
	Mean (SD)	Mean (SD)			Mean (SD)			Mean (SD)			
Knowledge of transmission (maximum possible score=6)	3.6 (1.6)	2.6 (1.7)			3.4 (2.0)			3.2 (1.8)			**<0.001**
Knowledge of signs and symptoms (maximum possible score=5)	4.9 (0.2)	4.9 (0.2)			4.9 (0.2)			4.9 (0.2)			0.916
Knowledge of prevention and treatment (maximum possible score=8)	6.4 (0.9)	5.8 (1.2)			5.9 (1.3)			6.0 (1.2)			**0.004**
Overall Knowledge of monkeypox (maximum possible score=21)	16.9 (2.3)	15.3 (2.3)			16.3 (2.5)			16.1 (2.5)			**<0.001**
**Perception of mpox**		**Jigawa (N=80)**		**Lagos (N=83)**			**Oyo (N=76)**				**Total (N= 239)**
		n (%)		n (%)			n (%)				n (%)
**Perceived susceptivity about monkeypox**											
I cannot contract monkeypox	Disagree	43 (53.8)		40 (48.2)			30 (39.5)				113 (47.3)
It does not affect people in my locality	Disagree	43 (53.7)		63 (75.9)			53 (69.7)				159 (66.5)
People around me are not at risk of monkeypox	Disagree	39 (48.7)		55 (66.3)			34 (44.7)				128 (53.5)
I am immune to monkeypox	Disagree	41 (51.3)		44 (53.0)			61 (80.3)				146 (61.1)
monkeypox does not affect children	Disagree	59 (73.8)		72 (86.8)			63 (82.9)				194 (81.2)
monkeypox is severe in people with chronic health condition	Agree	69 (86.3)		57 (68.7)			62 (81.6)				188 (78.6)
I may get monkeypox by having sexual intercourse with infected person	Agree	64 (80.0)		68 (81.9)			66 (86.8)				198 (82.8)
It can affect anyone	Agree	79 (98.7)		81 (97.6)			73 (96.1)				233 (97.5)
**Variables-Perceived severity of monkeypox**											
I think monkeypox is a very serious disease	Agree	80 (100.0)		76 (91.6)			73 (96.1)				229 (95.8)
I will feel sick if I have monkeypox	Agree	80 (100.0)		80 (96.4)			72 (94.7)				232 (97.1)
Being infected will stop my daily activities	Agree	76 (95.0)		79 (95.2)			70 (92.1)				225 (94.1)
I may require hospitalization if I get monkeypox	Agree	76 (95.0)		81 (97.6)			73 (96.1)				230 (96.2)
I think I can die from monkeypox	Agree	62 (77.5)		39 (47.0)			32 (42.1)				133 (55.7)
Other members of my household will feel sick if I get monkeypox	Agree	69 (86.3)		69 (83.1)			68 (89.4)				206 (86.2)
**Variables-Perceived benefits of adherence to monkeypox preventive and control strategies**											
My household will be safer if I protect myself from monkeypox	Agree	79 (98.7)		78 (94.0)			74 (97.4)				231 (96.6)
I will not get monkeypox if I follow recommended precautionary measures	Agree	78 (97.5)		78 (94.0)			73 (96.0)				229 (95.8)
**Variables-perceived barriers to adherence monkeypox preventive and control strategies**											
Someone with monkeypox will be stigmatized	Agree	78 (97.5)		74 (89.2)			69 (90.8)				221 (92.5)
Adherence to the recommended protective measures against monkeypox is not easy	Agree	63 (78.7)		43 (51.8)			22 (28.9)				128 (53.5)
Use of hand sanitizer is difficult because it is expensive for me	Agree	38 (47.5)		13 (15.7)			5 (6.6)				56 (23.4)
**Variables-perceived self-efficacy towards monkeypox preventive and control strategies**											
I feel confident in my ability to stay safe from monkeypox	Agree	76 (95.0)		70 (84.3)			64 (84.2)				210 (87.9)
I can comfortably abide by prevention guidelines released by the government	Agree	78 (97.5)		78 (94.0)			73 (96.0)				229 (95.8)
**Perception scores**	Jigawa State		Lagos State			Oyo State				Total	
	Median (IQR)		Median (IQR)			Median (IQR)				Median (IQR)	
Perception of susceptibility (maximum possible score=8)	6 (1.5)		6 (2)			6 (2)				6 (2)	
Perception of severity (maximum possible score=6)	6 (1)		5 (1)			5 (1)				5 (1)	
Perception on benefits of adherence (maximum possible score=2)	2 (0)		2 (0)			2 (0)				2 (0)	
Perception on barriers to adherence (maximum possible score=3)	2 (1)		1 (1)			1 (1)				2 (1)	
Perception on self-efficacy (maximum possible score=2)	2 (0)		2 (0)			2 (0)				2 (0)	

^h^One-way analysis of variance (ANOVA)

**Unadjusted and multivariable logistic regression analysis of knowledge of monkeypox among primary health care workers:** community health workers (CHEW/CHO) had 70% lower odds of having good knowledge of mpox compared to nurses or midwives (aOR: 0.30, 95% CI 0.11-0.79; p = 0.01) after adjusting for state and gender (Table 3).

**Table 3:** unadjusted and multivariable logistic regression analysis of knowledge of monkeypox among primary healthcare workers

	Unadjusted ORs (95% CI)	P-value	Adjusted ORs (95% CI)	P-value
**Respondent' age**				
15-29 years	ref		ref	
30-44 years	1.74 (0.76 -3.99)	0.186	1.51 (0.65 -3.51)	0.334
45 years and above	0.64 (0.23 -1.73)	0.386	0.54 (0.19 -1.49)	0.237
**Level of education**				
Less than tertiary	ref		ref	
Tertiary/further	2.54 (0.69 -9.32)	0.158	2.48 (0.66 -9.34)	0.178
**Respondent ethnicity**				
Yoruba	ref		ref	
Hausa/Fulani	2.14 (0.99 -4.63)	0.053	1.84 (0.45 -7.42)	0.390
**Marital status**				
Single/Never married	ref		ref	
Married/Married before	1.13 (0.50 -2.54)	0.764	0.94 (0.41 -2.17)	0.894
**Religion**				
Christianity	ref		ref	
Islam	1.73 (0.82 -3.65)	0.147	1.49 (0.65 -3.43)	0.340
**Average monthly income**				
Less than 30,000	ref		ref	
30,000 and above	1.21 (0.60 -2.43)	0.576	1.13 (0.55 -2.29)	0.727
No response	1.57 (0.74 -3.32)	0.234	1.10 (0.43 -2.78)	0.832
**Cadre**				
Nurse/Midwife	ref		ref	
CHEW/CHO	0.30 (0.12 -0.78)	0.013	0.30 (0.11 -0.79)	0.014
Others	0.28 (0.10 -0.76)	0.012	0.29 (0.11 -0.80)	0.017

**Association between perception and selected respondents' characteristics:** healthcare workers generally believed monkeypox is a serious illness that anyone can contract. However, there were some variations in perception. Workers in Jigawa perceived the disease as severe most (p≤0.001). Those with higher education saw themselves as more susceptible (p=0.016) and reported fewer barriers to following preventive measures. Interestingly, despite some variations, most workers acknowledged the benefits of preventative strategies and expressed confidence in adhering to them. Factors associated with higher perceived barriers included states (p≤0.001), religion (p≤0.001) and gender (p=0.002) while factors associated with higher perceived severity included state (p≤0.001) and respondent's gender (p≤0.001) (Table 4).

**Table 4:** association between perception and selected respondents' characteristics (N=239)

Respondent variables		Perception of susceptibility		
		Mean (±SD)	Mean ranking	P-value
State	Jigawa	5.46 (1.43)	107.61	
	Lagos	5.78 (1.56)	124.72	
	Oyo	5.81 (1.57)	127.88	0.127*
Highest Level of Education	Secondary	4.58 (1.97)	74.29	
	Tertiary/further	5.74 (1.48)	122.41	0.016**
Religion	Christianity	5.70 (1.62)	123.23	
	Islam	5.66 (1.42)	116.50	0.442**
Gender	Male	5.72 (1.48)	119.69	
	Female	5.67 (1.54)	120.10	0.968**
Respondent variables		**Perception of Severity**		
		Mean (±SD)	Mean ranking	P-value
State	Jigawa	5.53 (0.81)	142.56	
	Lagos	5.10 (1.03)	108.75	
	Oyo	5.10 (1.07)	108.52	<0.001*β
Highest level of Education	Secondary	5.50 (0.90)	141.25	
	Tertiary/further	5.23 (1.00)	118.87	0.232**
Religion	Christianity	5.13 (1.08)	111.75	
	Islam	5.37 (0.88)	128.88	0.036**
Gender	Male	5.59 (0.78)	146.81	
	Female	5.13 (1.03)	110.80	<0.001**
Respondent variables		**Perception of benefits of adherence to monkeypox preventive and control strategies**		
		Mean (±SD)	Mean ranking	P-value
State	Jigawa	1.96 (0.24)	123.98	
	Lagos	1.87 (0.39)	115.51	
	Oyo	1.93 (0.29)	120.72	0.152*
Highest level of Education	Secondary	1.75 (0.62)	106.83	
	Tertiary/further	1.93 (0.29)	120.69	0.096**
Religion	Christianity	1.92 (0.32)	119.31	
	Islam	1.93 (0.31)	120.74	0.692**
Gender	Male	1.90 (0.29)	119.06	
	Female	1.93 (0.39)	120.32	0.764**
Respondent variables		**Perception of barriers to adherence to monkeypox preventive and control strategies**		
		Mean (±SD)	Mean ranking	P-value
State	Jigawa	2.23 (0.71)	162.96	
	Lagos	1.56 (0.82)	110.37	
	Oyo	1.26 (0.64)	85.27	<0.001*β
Highest level of Education	Secondary	2.00 (0.85)	142.33	
	Tertiary/further	1.67 (0.83)	118.81	0.222**
Religion	Christianity	1.46 (0.78)	102.44	
	Islam	1.93 (0.83)	138.92	<0.001**
Gender	Male	1.98 (0.88)	141.79	
	Female	1.59 (0.79)	112.52	0.002**
Respondent variables		**Perception of self-efficacy towards monkeypox preventive and control strategies**		
		Mean (±SD)	Mean ranking	P-value
State	Jigawa	1.92 (0.26)	129.15	
	Lagos	1.78 (0.46)	114.81	
	Oyo	1.80 (0.43)	116.02	0.064*
Highest level of Education	Secondary	1.75 (0.45)	108.50	
	Tertiary/further	1.84 (0.40)	120.60	0.340**
Religion	Christianity	1.79 (0.44)	115.82	
	Islam	1.87 (0.35)	124.50	0.118**
Gender	Male	1.83 (0.38)	121.93	
	Female	1.83 (0.45)	119.33	0.683**

## Discussion

In this study, we assessed the level of awareness, knowledge, and risk perception of healthcare workers in Kiyawa and Dutse LGAs of Jigawa State, Lagelu and Ibadan Southwest LGAs of Oyo State, and Ikorodu LGA of Lagos states about mpox. We analyzed socio-demographic factors that might have influenced a healthcare worker's perception and knowledge and found that although overall awareness was high, there were variations in perceptions and knowledge across the three states. Additionally, we noted low knowledge levels of signs and symptoms of mpox among the healthcare workers.

The findings of our study highlight a gap in the capacity of health workers in Nigerian primary health facilities. We found that the primary healthcare workers possessed a low level of knowledge, especially regarding transmission of mpox, and their main source of information was mass media, rather than their medical training or government and scientific sources. The suboptimal knowledge of mpox transmission among the healthcare workers requires attention by the public health authorities in Nigeria given this group plays vital role in disease prevention, early detection and alert system. Their inadequate knowledge may affect their ability to detect and report early suspected cases of mpox to relevant authorities or provide appropriate counselling to community members in terms of prevention [26]. According to the national guidelines on integrated disease surveillance and response, healthcare workers in health facilities are to report suspected mpox to LGA and then to the state epidemiologist for necessary actions.

The lack of knowledge about the availability of an mpox vaccine among primary health is not surprising, despite primary healthcare being the bedrock of routine vaccination programmes in Nigeria. It rather reflects inequity in global vaccine access as exemplified by the global COVID-19 vaccine roll-out [27]. While infectious diseases such as tuberculosis, measles, diarrhea and pneumonia have received deserved and adequate attention in terms of vaccine development, investment in vaccine development and roll-out for mpox and other emerging diseases like Ebola virus disease, Marburg virus, and Lassa fever [28], which typically affect lower-income countries, remains urgent to combat the treats from these diseases and achieve global health equity. The limited knowledge about the availability of the mpox vaccine also highlights broader challenges within Nigeria's healthcare system and underscores the need for improved communication channels, training programs, and knowledge dissemination strategies to ensure that health workers are well-informed about the latest developments in disease prevention and control. Bridging this knowledge gap is critical to strengthen health workers capacity to respond and manage misinformation among community members in case of localized outbreaks of mpox.

Interestingly, we found that a significant number of health workers expressed perceived low level of risk in contracting mpox despite acknowledging their lack of immunity and susceptibility to mpox by everybody. This reflects a hierarchy of risk perception among health workers. They believe that while mpox is generally transmissible, they are somehow less susceptible. This finding is also in keeping with the tripartite model of risk perception which stipulates that risk perception may be deliberate, affective, or experiential [29]. Notwithstanding, the positive affirmation may be due to their socio-cultural and religious beliefs, which play crucial roles in shaping people's perceptions and attitudes towards the disease. Moreover, it may be due to their self-efficacy in adherence to mpox preventive strategies as most healthcare workers alluded to being self-efficacious about preventive strategies for mpox. Previous studies have highlighted reduction in disease' risk perception with increasing self-efficacy [30,31]. We also found evidence of regional differences in perceived severity of mpox and perceived barriers to adherence to mpox preventive and control strategies among health workers in primary health facilities in Jigawa State compared to Oyo and Lagos states. The reason for this finding is unclear, but we hypothesized that this relates to the differences in health system capacities and socio-economic characteristics across the states. Jigawa state has experienced large scale outbreaks of infectious diseases notably cerebrospinal meningitis and cholera which had overwhelmed the health system compared to Lagos state with a more responsive health system towards disease outbreak [32].

We had two key limitations in the study, the first being that we did not obtain data from healthcare workers in private, secondary and tertiary levels health facilities. Secondly, because of the convenient sampling, health workers that are not present at the facilities at the time of recruitment may have opinions different from those met at the facilities. Multiple outcomes were investigated, and therefore statistical findings may be the result of multiple hypothesis testing. The generalizability of the study findings is thus limited to primary healthcare settings. This study's strength lies in its comprehensive examination of healthcare worker knowledge and perception of monkeypox. It delves deeper than awareness by pinpointing knowledge gaps, especially in transmission routes. By revealing mass media as a concerning information source, the study emphasizes the need for improved training and communication channels. Exploring the risk perception among healthcare workers, the analysis considers the potential influence of self-efficacy and socio-cultural factors. Furthermore, regional variations shed light on the possible impact of health system capacity and socio-economic disparities. These valuable insights pave the way for targeted interventions to improve knowledge, combat misinformation, and ultimately enhance healthcare worker capacity in managing monkeypox.

## Conclusion

HCWs play a critical role in not only clinical care in outbreaks but also public health education and disease control, but their knowledge of mpox - an emerging disease of concern, was limited. To ensure that healthcare workers are equipped with accurate and evidence-based knowledge, efforts need to be made by medical schools and NCDC to strengthen information spread through training programmes, conferences, peer-reviewed publications, and collaboration among healthcare institutions and professional organizations. These initiatives will help enhance the expertise and competencies of HCWs, enabling them to provide the better care and guidance to patients and their communities.

### What is known about this topic

- *Monkeypox disease is considered rare and self-limiting;*
- *Monkeypox disease can be severe.*

### What this study adds

- *Health workers expressed perceived low level of risk in contracting mpox despite acknowledging their lack of immunity and susceptibility;*
- *Knowledge of mpox transmission is low compared to knowledge of signs/symptoms and knowledge of prevention and treatment.*
